# Neural ‘Bubble’ Dynamics Revisited

**DOI:** 10.1007/s12559-013-9214-3

**Published:** 2013-03-28

**Authors:** Paul C. Bressloff, Stephen Coombes

**Affiliations:** 1 Department of Mathematics, University of Utah, Salt Lake City, Utah 84112 USA; 2 Department of Mathematical Sciences, Center for Mathematical Medicine and Biology, University of Nottingham, Nottingham, NG7 2RD UK

**Keywords:** Neural field models, Self-organisation, Bumps, Breathers

## Abstract

In this paper, we revisit the work of John G Taylor on neural ‘bubble’ dynamics in two-dimensional neural field models. This builds on original work of Amari in a one-dimensional setting and makes use of the fact that mathematical treatments are much simpler when the firing rate function is chosen to be a Heaviside. In this case, the dynamics of an excited or active region, defining a ‘bubble’, reduce to the dynamics of the boundary. The focus of John’s work was on the properties of radially symmetric ‘bubbles’, including existence and radial stability, with applications to the theory of topographic map formation in self-organising neural networks. As well as reviewing John’s work in this area, we also include some recent results that treat more general classes of perturbations.

## Reflections

### **PCB**

 I first met John in 1985 when he interviewed me for a PhD. position in string theory at King’s College London. I had drifted away from academia at the time and was thus grateful that he decided to offer me the position. I was immediately struck by his sharpness, his larger-than-life personality, and his enthusiasm for mathematics and science. Although that enthusiasm would sometimes lead John to follow rather controversial paths, it was inspiring for a young graduate student. When I started my PhD, John had a large group of students working on horrendous perturbation calculations in supergravity. I still remember the long line of students disappearing down the corridor waiting to discuss their latest results. However, all of these students graduated during my first year so I was essentially John’s only student, which meant that we worked closely together. My thesis work formed part of a book on ‘Finite Superstrings’ [49] co-authored with John and one of his long-standing collaborators Alvarez Restuccia from Venezuela. After graduating, I joined the Long-range Research Lab at the Hirst Research Center in London, which carried out fundamental research for GEC-Marconi. I was asked to develop a research programme in neural networks, which coincidentally was a research area that John had embraced in the 1970s and returned to in the late 1980s. John became a research consultant for GEC-Marconi and this enabled us to continue collaborating. Unfortunately, John and I lost contact when I joined Loughborough University in 1993. In fact, I only saw John one more time, which was as a guest speaker at his ‘retirement’ colloquium. Of course, John remained as active as ever following his retirement, in particular, pursuing his lifetime fascination with the problem of consciousness.

### **SC**

 I was a PhD student with John from 1991–1994 at King’s College London together with a large cohort of other students, including Dan Allen, Neil Taylor, Rasmus Petersen, Paulo Adeodato, Bart Krekelberg, Oury Monchi, and Simon Hastings. My work at that time was concerned with investigating the computational ability of higher-order neurons (artificial single neuron models that could compute polynomial functions of their input) and recurrent networks of Hopfield type. This latter work involved statistical mechanical calculations of storage capacity, as well as the development of learning rules. John strongly encouraged me to think about the relevance of this work to hippocampal processing, which set me on the path to investigate the dynamics of biological, as opposed to artificial, neural networks. Since then, I have never looked back! I have very fond memories of my time at King’s, including the realisation within a few days of starting my PhD on neurocomputing that John was none other than the author of ‘Black Holes: The End of the Universe?’ [47], a popular science book that I had enjoyed reading as a teenager. I became aware that I was joining his group at a time when his research activity was moving primarily from topics in mathematical physics to the mathematical theory of neural networks. Although I never worked with John on neural field models and ‘bubble’ dynamics, it is a pleasure to describe, with Paul, some of his work on this topic as well as more recent progress in this area. It is a rare treat to have met someone as vibrant as John and with such broad-ranging scientific interests. I will miss his mellifluous tones.

## Introduction

In this paper, we revisit a problem that John considered in 1999 [48], namely the existence and stability of radially symmetric spots in two-dimensional (2D) neural fields, also known as ‘neural bubble dynamics’. Neural fields represent the large-scale dynamics of spatially structured networks of neurons in terms of nonlinear integrodifferential equations, whose associated integral kernels represent the spatial distribution of neuronal synaptic connections. One type of solution that emerges in the presence of nonlocal synaptic inhibition (lateral inhibition) is a stationary spot solution, also known as an activity bump. Such bumps are typically coexistent with a stable low-activity state (bistability) so that an initial stimulus is needed in order to transition from the low-activity state to the bump. However, the bump persists after removal of the stimulus, so that the bump represents a persistent spatially localised activity state [12, 24, 33]. One of the reasons why persistent activity bumps are of interest is that they are thought to arise in cortical circuits performing certain spatial working memory tasks. Working memory involves cortical “memory neurons” that establish a representation of a stimulus that persists after the stimulus is removed. A typical experiment is a delayed response task, in which a primate is required to retain information regarding the location of a sensory cue across a delay period between the stimulus and behavioural response. Physiological recordings in prefrontal cortex have shown that spatially localised groups of neurons fire during the recall task and then stop firing once the task has finished [22]. The stimulus response of a cell is characterised by a smooth tuning curve that is peaked at a preferred spatial cue and varies from cell to cell. At the network level, the memory of cue location is stored as an activity bump. Persistent activity bumps occur in a number of other systems that encode directional or spatial information, including head direction cells in thalamus and basal ganglia [46] and place cells in the hippocampus [38].

Prior to John’s study [48], almost all analyses of bumps had been restricted to 1D networks. Wilson and Cowan established the existence of 1D bumps numerically [53, 54], and Amari subsequently constructed an exact bump solution for a 1D scalar neural field equation with a Heaviside firing rate function [1]. He also showed how the stability of the bump depends on whether or not perturbations of the bump boundary (threshold crossing points) grow or decay. Interestingly, Amari also developed an analysis of 2D bumps. However, this work only appeared in a 1978 book that was never translated from the Japanese [3] and has only just been summarised in English [5]. John generalised Amari’s 1D analysis by deriving conditions for the existence of radially symmetric 2D bumps and by determining the stability of the bumps with respect to radially symmetric perturbations of the circular bump boundary [48], see also [50]. This analysis was later extended to the case of nonradially symmetric perturbations using Fourier methods and properties of Bessel functions [16, 20, 21, 25, 39]. In related work, Laing and Troy [32] introduced PDE methods to study symmetry breaking of rotationally symmetric bumps and the formation of multiple bump solutions. However, such PDE methods can only be applied to specific forms of weight distribution. In particular, they break down if the weight distribution has compact support.

In section ‘Dynamics of a ‘Bubble’ Boundary’, we review the work of John (in an appropriately revised notation) on 2D bumps, as well as describe how to treat azimuthal instabilities, and the generation of breathing bumps in neural field models with adaptation and external drive. Next in section ‘Self-Organising Neural Field Theory’ we cover John’s work on how 2D bump activity can drive topographic map formation in self-organising neural networks, also presented in [48], and describe a further set of techniques for treating nonradially symmetric perturbations. We end with a brief discussion in section ‘Discussion’.

## Dynamics of a ‘Bubble’ Boundary

Consider the following neural field equation for a 2D sheet of neural tissue

1 $$ \tau \frac{\partial{u({\bf r},t)}}{\partial t}=-u({{\bf r}},t)+\int\limits_{{\mathbb{R}}^2}w(|{\bf r}-{\bf r}^\prime|) f(u({\bf r}^\prime,t)) {\rm d} {\bf r}^\prime $$

where **r** = (*r*, *θ*) and **r**′ = (*r*′, *θ*′). The neural field *u*(**r**, *t*) represents the local activity of a population of neurons at position **r**, *τ* is a synaptic or membrane time constant (which we set to unity), *w* is the distribution of synaptic weights, and *f* denotes an output firing rate function. The weight distribution is assumed to depend on the Euclidean distance |**r** − **r**′| between interacting neurons at **r** and **r**′. A common choice for the firing rate function is a bounded, monotonic function such as a sigmoid. Here we follow Amari [1] and Taylor [48] and take this to be Heaviside function with threshold* κ* such that *f*(*u*) = *H*(*u* − *κ*). Below we review John’s extension of Amari’s 1D analysis to radially symmetric localised solutions in 2D. For a more detailed discussion of methods for analysing the existence and stability of bumps in neural fields, see recent reviews by the authors [9, 14].

First let us rewrite the original model (1) in the form *u*_*t*_ =  −*u* +* ψ*, where

2 $$ \psi({\bf r},t)=\int\limits_{{\mathcal{B}}({\bf r}^\prime,t)} {\rm d} {\bf r}^\prime w(|{\bf r}-{\bf r}^\prime|), $$

and $\mathcal{B}({\bf r},t)=\{{\bf r} | u({\bf r},t) \geq \kappa\}$ (which defines the ‘bubble’). For radially symmetric spot solutions of radius $\Updelta(t)$ then we have that* ψ*(**r**, *t*) = *ψ*(*r*, *t*) with

3 $$ \psi(r,t)= \int\limits_0^{2 \pi} {\rm d} \theta \int\limits_0^{\Updelta(t)} w \left (\sqrt{r^2+{r^\prime}{^2} - 2 r r^\prime \cos \theta} \right ) r^\prime {\rm d} r^\prime. $$

Here $\Updelta(t)$ is defined by the level set condition $u(\Updelta(t),t)=\kappa$. Time-independent spot solutions such that $\Updelta(t)=\Updelta$ (namely with a constant radius) such that *u*(**r**, *t*) = *U*(*r*) with $\lim\nolimits_{r \rightarrow \infty}U(r)=0$ and $U(r) \gtrless \kappa$ for $r \lessgtr \Updelta$ are given by

4 $$ U(r) = \left. \psi(r,t) \right |_{\Updelta(t)=\Updelta}. $$

Differentiating the level set condition with respect to time gives an equation for the velocity of the spot boundary in the form

5 $$ \frac{{\rm d} {\Updelta}}{{\rm d} {t}} = - \left. \frac{\partial u(r,t) /\partial t}{\partial u(r,t) /\partial r} \right |_{r=\Updelta}. $$

Using (1) we may derive an ODE for $v=\left. \partial u(r,t) /\partial r \right |_{r=\Updelta}$ as

6 $$ \frac{{\rm d} {v}}{{\rm d} {t}} = -v +\left. \frac{\partial {\psi}}{\partial {r}} \right |_{r=\Updelta}. $$

Hence, we may generate a system of two exact nonlinear ODEs for $(\Updelta,v)$ to describe the evolution of the (radially symmetric) spot:

7 $$ \frac{{\rm d} {\Updelta}}{{\rm d} {t}} = - \frac{-\kappa+\Uppsi(\Updelta)}{v}, \quad \frac{{\rm d} {v}}{{\rm d} {t}} = -v +\Uppsi^\prime(\Updelta), $$

where

8 $$ \Uppsi(\Updelta) = \int\limits_0^{2 \pi} {\rm d} \theta \int\limits_0^\Updelta w\left(\sqrt{\Updelta^2+r^2-2\Updelta r \cos \theta}\right) r {\rm d} r. $$

The steady state of (7) is determined by the solution of $\Uppsi(\Updelta)=\kappa$ and $v=\Uppsi'(\Updelta)$. A linear stability analysis around the steady-state solution shows that there are two eigenvalues, one with value −1 (that can not lead to any instability) and the other with a value λ, where

9 $$ \lambda = -\Uppsi^\prime(\Updelta)/v. $$

Hence, only the solution with $\Uppsi^\prime(\Updelta)<0$ is stable (after realising that *v* is the gradient of the spot at its boundary and is negative).

In the ‘Appendix’, we show that *U*(*r*) can be written in the computationally useful form

10 $$ U(r)= 2 \pi \Updelta \int_0^{\infty} \widehat{w}(\rho) J_0( \rho r) J_1( \rho \Updelta) {\rm d} \rho, $$

where $\widehat{w}$ is the 2D Fourier transform of *w*. For the sake of illustration, consider a wizard hat weight distribution given by a combination of modified Bessel functions of the second kind

11 $$ w(r) = \frac{2}{3 \pi}\left( K_0(r) - K_0(2r) - A(K_0(r/\sigma) - K_0(2r/\sigma)) \right). $$

**Fig. 1:**
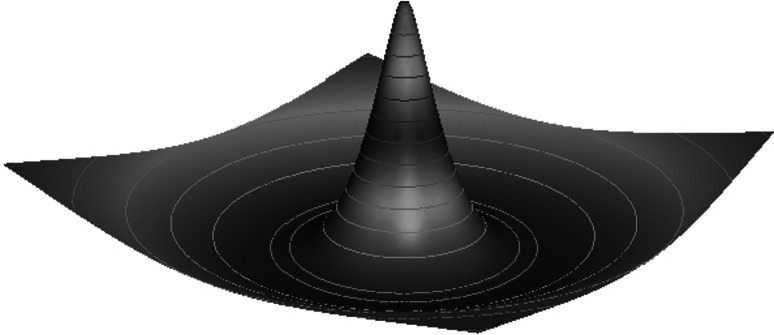
A* plot* of the weight distribution describing short-range excitation and long-range inhibition. The form of *w*(**r**) given by (11) with *A* = 3/4 and* σ* = 4 generates a two-dimensional wizard hat function

As shown in Fig. 1, this can generate a weight distribution with short-range excitation and long-range inhibition. Using the fact that the corresponding 2D Fourier transform of *K*_0_(*s* *r*) is ${\mathcal H}( \rho, s) = (\rho^2 + s^2)^{-1}$, we have

12 $$ \widehat{w}(\rho) = \frac{2}{3 \pi} ({\mathcal H}(\rho,1) - {\mathcal H}(\rho,2) - A({\mathcal H}(\rho,1/ \sigma) - {\mathcal H}(\rho, 2/ \sigma))). $$

Thus, the integral (10) can be evaluated explicitly using the identity

13 $$ \int\limits_0^{\infty} \frac{J_0(\rho r) J_1(\rho \Updelta )}{\rho^2 + s^2} {\rm d} \rho \equiv {\mathcal I}(\Updelta,r,s) = \left\{ \begin{array}{ll} \frac{1}{ s} I_1(s \Updelta) K_0(s r) & r > \Updelta \\ \frac{1}{ s^2\Updelta} - \frac{ 1}{ s} I_0( s r ) K_1 (s \Updelta) & r<\Updelta, \end{array}\right. $$

where *I*_*ν*_ is the modified Bessel function of the first kind of order* ν*. Thus, the stationary bump *U*(*r*) has the form

14 $$ U(r) = \frac{4\Updelta}{3 } \left [ {\mathcal I}(\Updelta,r,1) - {\mathcal I}(\Updelta,r,2) -A( {\mathcal I}(\Updelta,r,1/ \sigma) - {\mathcal I}(\Updelta,r,2/ \sigma)) \right]. $$

The bump radius may then be computed by finding the roots $\Updelta$ of the equation $\kappa = \Uppsi (\Updelta)$ with

15 $$ \begin{aligned} \Uppsi(\Updelta) &= \frac{4\Updelta}{3} \left( I_1(\Updelta) K_0(\Updelta) - \frac{1}{2} I_1(2\Updelta) K_0(2\Updelta) \right.\\ &\quad\left.- A( \sigma I_1(\Updelta/ \sigma) K_0(\Updelta/ \sigma) - \frac{\sigma}{2} I_1(2\Updelta/\sigma) K_0(2\Updelta \sigma) ) \right). \end{aligned} $$

**Fig. 2:**
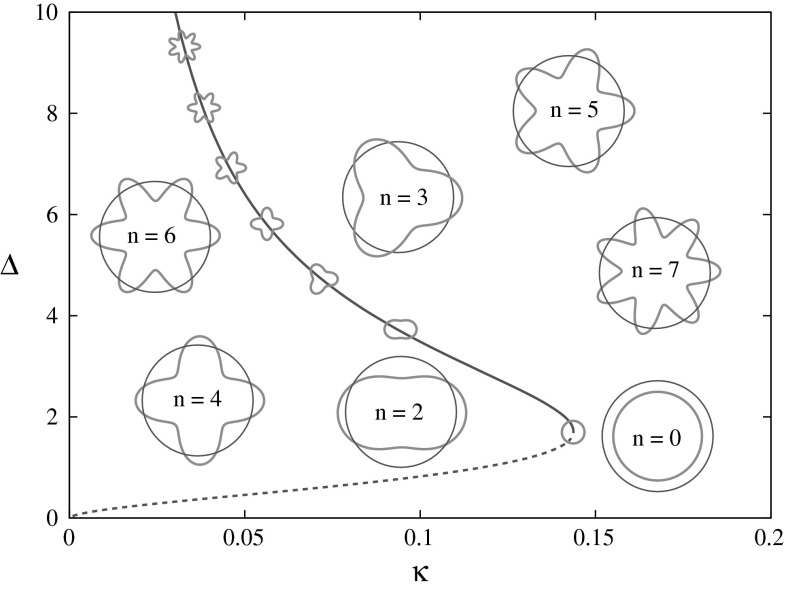
The bump width $\Updelta$ as a function of threshold* κ*, for the wizard hat weight distribution (11) with *A* = 1/4 and* σ* = 2, is shown by the continuous* dark blue line*. According to the linear radial instability analysis, the* solid* (*dashed*)* line* indicates a stable (unstable) branch of solutions. The azimuthal instability analysis predicts that solutions on the upper branch can also develop instabilities as the threshold is decreased leading to the formation of solutions exhibiting *D*_*n*_ symmetry. The points of azimuthal instability are marked by glyphs in the shape of the corresponding mode (Color figure online)

Note that the threshold condition is a necessary but not sufficient condition for proving existence of a 2D bump. One also has to check that there are no other threshold crossing points. In the case of a Mexican or wizard hat weight distribution, there is typically a maximum of two spot solutions as illustrated in Fig. 2 for *w* given by Eq. (11). Using (9), we find that the narrower spot is always unstable as found in 1D. However, the stable upper branch can develop instabilities as the threshold is decreased leading to the formation of solutions that break the rotational symmetry [32, 39], see below.

### Azimuthal Instabilities

It can be misleading to extrapolate results about bumps in 1D to spots in 2D. For example, it was known to Amari [1] in his seminal work on 1D models with Mexican hat weight distribution that bump solutions come in pairs, and that it is only the wider of the two that is stable. Extrapolating this to 2D, and focusing on radially symmetric spots, one would conclude a similar state of affairs for radial perturbations to the stationary spot, as done by John [48]. However, in the 2D setting one must also pay careful attention to azimuthal instabilities. This has been appreciated by a number of authors in recent years [20, 32, 39], though anticipated much earlier by Amari [3]. Indeed azimuthal instabilities can destabilise spots on the wide branch (that are stable to radial perturbations) and lead to the generation of intricate labyrinthine structures [16, 39].

In order to determine linear stability of a bump solution *U*(*r*), substitute *u*(**r**, *t*) = *U*(*r*) + *p* (**r**)e^*λt*^ into Eq. (1) and expand to first order in *p* using equation (4). This gives the eigenvalue equation

16 $$ \begin{aligned} (\lambda+1)p({{\bf r}})&= \int w(|{{\bf r}} - {{\bf r}}^\prime|) \delta(U(r^\prime) -\kappa))p({{\bf r}}'){\rm d} {{\bf r}}^\prime\\ &=\frac{1}{|U^\prime(\Updelta)|} \int\limits_0^{\infty}\int\limits_0^{2\pi} w(|{{\bf r}}-{{\bf r}}^\prime|)\delta(r^\prime-a)p({{\bf r}}^\prime){\rm d} \theta^\prime r^\prime {\rm d} r^\prime \\ &=\frac{\Updelta}{|U^\prime(\Updelta)|}\int\limits_0^{2\pi} w(|{{\bf r}}-{{\bf a}}^\prime|)p(a,\phi^\prime){\rm d} \phi^\prime, \end{aligned} $$

where **a**′ = (*a*, *ϕ*′). We can now formulate the stability problem in terms of finding the spectrum of a compact linear operator acting on continuous, bounded functions* ψ*(*r*, *ϕ*) defined on the disc of radius $r\leq \Updelta$. One class of solution to Eq. (16) consists of functions *p*(**r**) that vanish on the boundary,* ψ*(*a*,*ϕ*) = 0 for all* ϕ*, such that* λ* =  −1. This belongs to the essential spectrum, which does not contribute to any instabilities. The discrete spectrum is determined by setting ${{\bf r}}={{\bf a}}\equiv (\Updelta,\phi)$ in Eq. (16):

17 $$ (\lambda+1) p(\Updelta, \phi) = \frac{\Updelta}{|U'(\Updelta)|} \int\limits_{0}^{2\pi} w \left( 2 \Updelta \sin\left ( \frac{\phi - \phi'}{2} \right )\right) p(\Updelta, \phi') {\rm d} \phi', $$

where we have simplified the argument of *w*(*r*) using

18 $$ |{{\bf a}}-{{\bf a}}'| =\sqrt{2\Updelta^2-2\Updelta^2\cos(\phi-\phi^\prime)} = 2\Updelta\sin \left (\frac{\phi - \phi'}{2}\right ). $$

Equation (17) can be solved in terms of Fourier eigenmodes, that is, $p(\Updelta,\phi)=P_n(\phi)=c_n{\rm e}^{in\phi}+\overline{c}_n{\rm e}^{-in\phi}$ with corresponding eigenvalue* λ*_*n*_ satisfying

19 $$ \lambda_n=-1+\frac{\Updelta}{|U^\prime(\Updelta)|} \int\limits_0^{2\pi}w(2\Updelta\sin(\phi/2)){\rm e}^{-in\phi}{\rm d} \phi. $$

Note that* λ*_*n*_ is real since (after rescaling* ϕ*)

20 $$ {\rm Im}\{\lambda_n\}=-\frac{2\Updelta}{|U^\prime(\Updelta)|}\int\limits_0^\pi w(2\Updelta\sin(\phi))\sin(2n\phi){\rm d} \phi=0, $$

i.e., the integrand is odd-symmetric about π/2. Hence,

21 $$ \lambda_n ={\rm Re} \{\lambda_n\}= -1+\frac{\Updelta}{|U^\prime(\Updelta)|}\int\limits_0^{2\pi} w(2\Updelta\sin(\phi/2))\cos(n\phi){\rm d} \phi, $$

with the integrand even-symmetric about* π*/2. The Fourier eigenmodes *P*_*n*_(*ϕ*) can be related to perturbations of the bump boundary. That is, if *u*(**r**, *t*) = *U*(*r*) + *ɛ* *p*(**r**, *t*) = 0 at ${{\bf r}}\equiv (r,\phi)=(\Updelta+\varepsilon a(\phi,t),\phi)$, where* ɛ* *a*(*ϕ*, *t*) with* ɛ* ≪ 1 denotes a small perturbation of the circular bump boundary at polar coordinate $(\Updelta,\phi)$ at time *t*, then

22 $$ \begin{aligned} \kappa &= u(\Updelta + \varepsilon a( \phi,t),\phi,t) = U(\Updelta +\varepsilon a(\phi,t)) +\varepsilon p(\Updelta +\varepsilon a(\phi,t),\phi,t),\\ &\approx U(\Updelta) + \varepsilon U^\prime(\Updelta) a ( \phi,t) +\varepsilon p (\Updelta, \phi,t). \end{aligned} $$

Since $U(\Updelta)=\kappa$, it follows that

23 $$ a( \phi,t) \approx \frac{p(\Updelta, \phi, t)}{|U'(\Updelta)|}. $$

Thus, one can decompose time-dependent perturbations of the circular boundary in terms of the Fourier modes [*c*_*n*_e^*in*ϕ^ + *c*_*n*_e^−*in*ϕ^]e^λ *t*^. Some examples of perturbations of the bump boundary are shown as insets in Fig. 2. It can be seen that the *n*th-order boundary perturbation has *D*_*n*_ symmetry, meaning the resulting solution has the *n* reflectional and rotational symmetries of the dihedral group *D*_*n*_. The perturbations also have a simple geometric interpretation. For example, *n* = 0 corresponds to a uniform expansion or contraction of the spot, whereas *n* = 1 corresponds to a uniform shift of the spot.

Since the *n* = 1 mode represents pure shifts of the spot solution, we expect* λ*_1_ = 0 from translation symmetry. In order to verify this, we evaluate the integral appearing in equation (21) using Bessel functions, along similar lines to the evaluation of *U*(*r*), Eq. (10). That is,

24 $$ \begin{aligned} \int\limits_0^{2\pi}w(|{{\bf a}}-{{\bf a}}^\prime|)\cos(n\phi^\prime){\rm d} \phi^\prime&=\int\limits_0^{2\pi}\left(\int\limits_0^\infty \widehat{w}(\rho)J_0(\rho |{{\bf a}}-{{\bf a}}^\prime|)\rho {\rm d} \rho\right) \cos\phi^\prime {\rm d} \phi^\prime \\ &=2 \pi \int\limits_0^{\infty} \widehat{w}(\rho) J_n( \rho \Updelta) J_n( \rho \Updelta) \rho {\rm d} \rho. \end{aligned} $$

Moreover, differentiating Eq. (10) with respect to *r* gives

25 $$ U'(\Updelta) =-{2 \pi \Updelta} \int\limits_0^{\infty} \widehat{w} (\rho) J_1 (\Updelta \rho) J_1 (\Updelta \rho)\rho {\rm d} \rho. $$

Hence, the eigenvalue (21) can be rewritten as

26 $$ \lambda_n=-1+\frac{\int_0^{\infty} \widehat{w}(\rho) J_n( \rho r) J_n( \rho \Updelta) \rho {\rm d} \rho} {\int_0^{\infty} \widehat{w}(\rho) J_1( \rho r) J_1( \rho \Updelta) \rho {\rm d} \rho}. $$

**Fig. 3:**
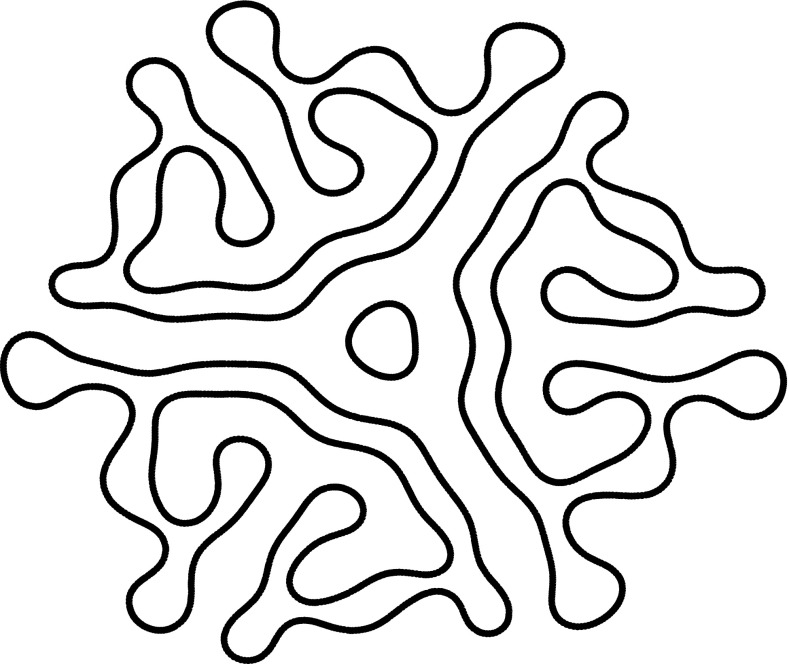
Beyond the point of an azimuthal instability, direct numerical simulations of the neural field model show the development of intricate labyrinthine structures from an initial spot solution. Here we show the shape of such a structure at a fixed moment in time. As the pattern evolves, it fills more of the spatial domain. The* black lines* denote the borders between high and low firing regions. This example illustrates the type of pattern that one can expect to see beyond an *n* = 3 azimuthal instability. For further examples and discussion, see [16]

It immediately follows that* λ*_1_ = 0. Hence, the 2D spot is linearly stable if λ_*n*_ < 0 for all *n* ≠ 1. For the weight distribution (11), the points of azimuthal stability as determined by the conditions* λ*_*n*_ = 0 can be calculated as [16]:

27 $$ 1=\frac{\sum_{i=1}^4 A_i K_n(\alpha_i \Updelta) I_n(\alpha_i \Updelta)}{\sum_{i=1}^4 A_i K_1(\alpha_i \Updelta) I_1(\alpha_i\Updelta)}, $$

where (*A*_1_, *A*_2_, *A*_3_, *A*_4_, α_1_, α_2_, α_3_, α_4_) = (1,  − 1,  − *A*, *A*, 1, 2, 1/σ, 2/σ). In Fig. 2 we plot the set of points for *n* = 0, 2 , …, 9 as determined by equation (27) to illustrate how the upper branch of rotationally symmetric spots can become unstable as the threshold is decreased, leading to the formation of solutions that breaks continuous rotational symmetry. The discrete rotational symmetry *D*_*n*_ of a bifurcating solution reflects the order *n* of the dominant eigenvalue* λ*_*n*_ at bifurcation. Interestingly, if linear adaptation is included in the neural field model, then these nonrotationally symmetric solutions can undergo a secondary instability leading to the formation of a rotating wave [32, 39]. Sufficiently strong adaptation can also destabilise a bump leading to a travelling spot [15, 16]. An example of the shape of an expanding labyrinthine structure that can emerge when a spot destabilises to an *n* = 3 mode is shown in Fig. 3.

### Stimulus-Induced Breathers

So far we have focused on activity bumps that persist in the absence of external stimuli due to the combined action of local recurrent excitation and lateral inhibition. We now describe some interesting instabilities that arise in the case of nonpersistent bumps [20, 21]. For the sake of illustration, consider a 2D excitatory neural field with linear adaptation and an external input *I*:

28 $$ \begin{aligned} \frac{\partial u ({{\bf r}},t)}{\partial t} &= -u({{\bf r}},t) + \int\limits_{{\mathbb{R}}^2} w(|{{\bf r}} - {{\bf r}}^\prime|) H(u({{\bf r}}^\prime,t)-\kappa) {\rm d} {{\bf r}}^\prime -\beta v({{\bf r}},t)+I({{\bf r}})\\ \frac{1}{\epsilon}\frac{\partial v ({{\bf r}},t)}{\partial t} &=-v({{\bf r}},t)+u({{\bf r}},t), \end{aligned} $$

where *v*(**r**, *t*) represents a local negative feedback mechanism, such as spike-rate adaptation, with* ε*, *β* determining the relative rate and strength of feedback [41]. Suppose that the inhomogeneous input is a radially symmetric Gaussian centred about the origin, $I({{\bf r}})=I_0\hbox{e}^{-r^2/\sigma_s^2}$. In the absence of an input, the resulting excitatory network supports travelling waves rather than stationary bumps. On the other hand, for sufficiently strong input amplitude *I*_0_, the network supports a radially symmetric bump centred about the input. Such a bump is not persistent, since if the input is removed then the bump disappears as well. The basic problem we want to address is what happens to the stability of the bump as the input amplitude is slowly decreased.

The analysis of the existence and stability of radially symmetric 2D bumps proceeds as above with minor changes. First, the threshold condition for the existence of a bump becomes

29 $$ \kappa= U(\Updelta)=2 \pi \Updelta \int\limits_0^{\infty} \widehat{w}(\rho) J_0( \rho \Updelta) J_1( \rho \Updelta) {\rm d} \rho + I(\Updelta). $$

Second, the linear stability of the bump is determined by the pair of eigenvalues* λ* = *λ*_*n*_^ ± ^ associated with the Fourier modes [*c*_*n*_e^*inϕ*^ + *c*_*n*_e^−*inϕ*^]e^λ *t*^, where [20]

30 $$ \lambda_n^{\pm}=\frac{1}{2}\left [-\Uplambda_n \pm \sqrt{\Uplambda_n^2-4\epsilon(1+\beta)(1-\Upgamma_n)}\right ], $$

31 $$ \Uplambda_n=1+\epsilon -\Upgamma_n(1+\beta),\quad \Upgamma_n =\frac{\mu_n(\Updelta)}{|U'(\Updelta)|(1+\beta)}, $$

and

32 $$ \mu_n(\Updelta)= \Updelta \int\limits_0^{2\pi}w(2a\sin( \phi/2))\cos(n\phi) {\rm d} \phi. $$

It follows that stability of a radially symmetric bump require $\Uplambda_n > 0$ and $\Upgamma_n < 1$ for all *n* ≥ 0. Given the form of $\Uplambda_n$, this reduces to the following stability conditions:

33 $$ \begin{array}{ll} \epsilon > \beta : \Upgamma_n < 1 & \hbox{for all}\,n\geq 0\\ \epsilon < \beta : \Upgamma_n < {\frac{1+\epsilon}{1+\beta}}& \hbox{for all}\,n\geq 0. \end{array} $$

**Fig. 4:**
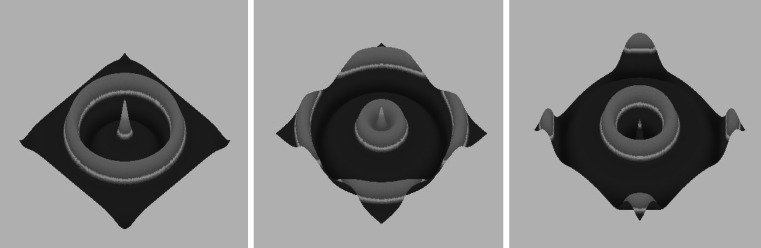
Sequence of snapshots of a 2D breather acting as a periodic pulse emitter in the case of a 2D excitatory neural field with linear adaptation and exponential weight function *w*(*r*) = e^−*r*^/(2* π*). Parameters are $\beta=4, \,\kappa=0.2,\,\epsilon=0.1$ and *I*_0_ = 0.2.* Lighter colours* indicate higher activity [20]

If the first condition is violated as some parameter is varied, then there is a saddle–node bifurcation, whereas a breakdown of the second condition signals a Hopf bifurcation. In the latter case, the bump instability leads to the formation of a breather. In the particular case of an excitatory network (such as obtained by setting *A* = 0 in Eq. (11)), with *w*(*r*) ≥ 0 for all *r* ≥ 0, we have

34 $$ \mu_n \leq \Updelta \int\limits_0^{2\pi} w(2\Updelta\sin(\phi/2))|\cos(n\phi)| {\rm d}\phi \leq \Updelta \int\limits_0^{2\pi} w(2\Updelta\sin(\phi/2)) {\rm d} \phi, $$

so that *μ*_*n*_ ≤  *μ*_0_ for all *n*. Since *μ*_1_ = 0, it follows that *μ*_0_ ≥ 0 and, hence, a purely excitatory neural field cannot support stable radially symmetric bumps. In this case, we expect any instability to involve the growth of radially symmetric perturbations, and hence, the resulting breather will be radially symmetric. On the other hand, if there is a Mexican hat weight distribution, then nonradially symmetric breather and rotating waves can occur [21, 39]. One way to induce a Hopf instability of a bump is to reduce the amplitude *I*_0_ of the Gaussian input; this modifies both the pulse-width $\Updelta$ and the slope of the bump at threshold, $|U'(\Updelta)|$. Interestingly, as the input amplitude is further reduced, the breather can undergo a secondary instability such that it now acts as an oscillating core that emits circular target waves. An example of such a periodic wave emitter is shown in Fig. 4. Thus, a spatially localised stationary input provides a mechanism for the formation of a network pacemaker oscillator. For a recent discussion of breathers in the absence of localised input, see [15].

## Self-Organising Neural Field Theory

One of the striking features of the visual system is that the visual world is mapped on to the cortical surface in a topographic manner. This means that neighbouring points in a visual image evoke activity in neighbouring regions of visual cortex. Superimposed upon this topographic map are additional maps reflecting the fact that neurons respond preferentially to stimuli with particular features such as ocular dominance and orientation [27]. In recent years, much information has accumulated regarding the two-dimensional distribution of both orientation preference and ocular dominance columns using optical imaging techniques [6, 7]. These experimental studies indicate that there is an underlying periodicity in the microstructure of V1 with a period of approximately 1 mm (in cats and primates). The fundamental domain of this tiling of the cortical plane is the hypercolumn, which contains two sets of orientation preferences* θ*  ∈ [0,*π*) per eye, organised around a pair of orientation singularities or pinwheels [37]. It is generally accepted that the preference of cortical neurons for particular stimulus features such as orientation and ocular dominance arises primarily from the spatial arrangement of convergent feedforward afferents from the LGN (or from other layers of cortex). Although molecular cues and axon guidance are involved in the initial stages of cortical map formation, the resulting maps are rather crude and some form of spontaneous or stimulus-driven activity appears to be necessary for the subsequent refinement of these maps through the pruning of initially exuberant axonal arborisations.

A large number of models have been proposed that describe activity–dependent development as a self-organising Hebbian process (see the review of Swindale [44]). In the case of correlation–based developmental models [35], the statistical structure of input correlations provides a mechanism for spontaneously breaking some underlying symmetry of the neuronal receptive fields leading to the emergence of feature selectivity. When such correlations are combined with intracortical interactions, there is a simultaneous breaking of translation symmetry across cortex leading to the formation of a spatially periodic cortical feature map. Correlation-based models are essentially linear, so that considerable insight into the developmental process can be obtained by solving an associated eigenvalue problem [36]. One of the possible limitations of this class of model is that a regular topographic map is assumed already to exist before feature-based columns begin to develop. In order to model the joint development of topography and cortical feature maps, it appears necessary to introduce some form of nonlinear competition for activation [30, 52], neurotrophic factors [17], or a combination of the two [51].

**Fig. 5:**
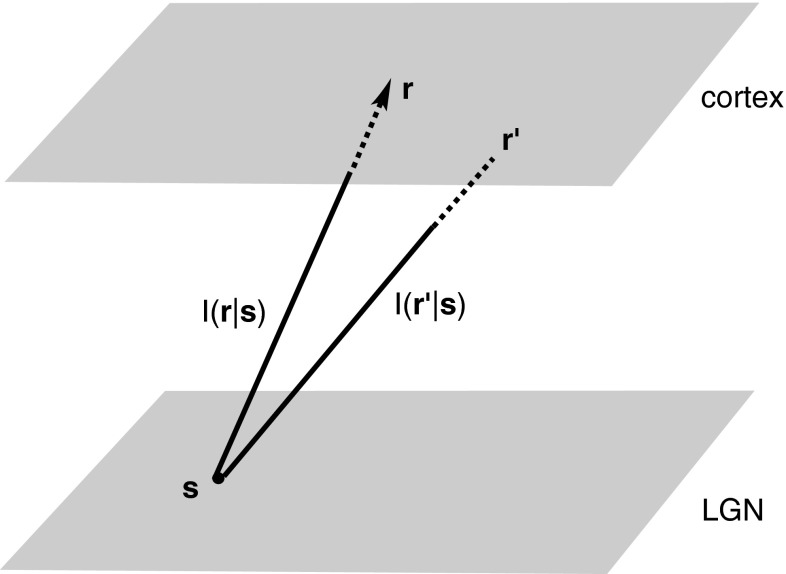
Basic network architecture illustrating an input pattern *I* from position **s** in the LGN layer that innervates the cortical layer at different positions **r**, **r**′

An alternative mathematical formulation of topographic map formation has been developed by Amari using the theory of self–organising neural fields [2, 4, 45]. At the simplest level, this model can be formulated in terms of a two-layer network corresponding respectively to the lateral geniculate nucleus (LGN) of the thalamus and the primary visual cortex (see Fig. 5). The cortical or output layer behaves pretty much as in the single-layer model given by equation (1). However, the inputs *I* are now determined by activity in the LGN or input layer together with the strength of the feedforward connections from the LGN to cortex. These connections are modifiable by experience via Hebbian learning. Initially, an activity pattern in the form of a bump occurs at some random location within the LGN and elicits a corresponding activity bump in cortex whose location depends on the given pattern of feedforward connections. Over timescales much slower than the relaxation time τ for cortical dynamics, inputs arising from many different locations within LGN (and hence the visual image) occur. The resulting set of cortical responses to this set of inputs then determines how the feedforward weights are modified. In the one-dimensional case, Amari [45] derived conditions for the existence and stability of a continuous topographic map between the two layers. Moreover, he showed that under certain circumstances, the continuous topographic map can undergo a pattern-forming instability that spontaneously breaks continuous translation symmetry, and the map becomes partitioned into discretised blocks; it has been suggested that these blocks could be a precursor for the columnar microstructure of cortex [45]. Given that cortical columns tend to be associated with stimulus features such as ocular dominance and orientation, this raises the interesting question whether or not such features could also emerge through the spontaneous symmetry breaking of self-organising neural fields. In the same paper on bubble dynamics [48], John extended Amari’s theory to 2D networks, again focusing on existence and stability with respect to radially symmetric perturbations. Stability with respect to a more general class of perturbations was subsequently analysed by Bressloff [8], who showed that stimulus preference maps could indeed emerge through the spontaneous symmetry breaking of self-organising neural fields. This analytical result was consistent with a number of numerical studies [19, 56]. In this section, we outline the basic steps in the analysis of 2D self-organising neural fields.

### Existence and Stability of Bumps

Let ${\bf s} \in \Upomega_2$ denote a point in the LGN layer and ${{\bf r}} \in \Upomega_1$ a point in the cortical layer, as shown in Fig. 5. Each point **s** labels a distinct input pattern which we denote by $I({{\bf r}}|{{\bf s}}), {{\bf r}} \in \Upomega_1$. Let *u*(**r**|**s**) denote the corresponding cortical activity induced by the input *I*(**r**|**s**), which is assumed to converge to a stable equilibrium given by the solution of the integral equation

35 $$ u({{\bf r}}|{{\bf s}})=\int\limits_{\Upomega_1} w(|{{\bf r}}-{{\bf r}}^\prime|)H(u({{\bf r}}^\prime|{{\bf s}})-\kappa){\rm d}{{\bf r}}' +I({{\bf r}}|{{\bf s}}). $$

Now suppose that once the network has reached equilibrium, a new input pattern is presented to the network from a different point **s**′, and that this process is iterated multiple times so that the network samples over the space of inputs. How the network responds to the given set of input patterns then determines how these input patterns are themselves modified by experience. Adaptation is introduced into the model by assuming that the input patterns evolve according to the dynamical equation

36 $$ \eta\frac{\partial I}{\partial t}=-I({{\bf r}}|{{\bf s}},t)+ \int\limits_{\Upomega_2} g(|{{\bf s}}-{{\bf s}}^\prime|) H(u({{\bf r}}|{{\bf s}}^\prime,t)-\kappa){\rm d} {{\bf s}}^\prime. $$

Here* η* ≫* τ* where* τ* is the relaxation time for cortical dynamics. Hence, $u({{\bf r}}|{{\bf s}},t),{{\bf r}}\in\Upomega_1$ denotes the equilibrium cortical state given by Eq. (35) in response to the input pattern *I*(**r**|**s**, *t*) for fixed **s**, *t*. The integral kernel *g* incorporates the effect of statistical correlations between activity patterns at different points in LGN. Thus, increasing the strength of an input from **s** also leads to an increase in the strength of an input at **s**′ provided that **s** and **s**′ are sufficiently close. Inputs from well-separated points in LGN are anticorrelated. These features can be included by taking *g* to be of the form

37 $$ g(|{{\bf r}}|)= c{\rm e}^{-{{\bf r}}^2/4\sigma^2} -\hat{c} $$

for constants *c*, *ĉ*.

Equations (35) and (36) are the basic neural field equations for topographic map formation [2, 45]. Rescaling the LGN and cortical coordinates appropriately, that is, ignoring the effects of cortical magnification, one can then look for homogeneous steady–state solutions of the form *u*(**r**|**s**) = *U*(|**r** − **s** |) and $I({{\bf r}}|{{\bf s}})=\overline{I}(|{{\bf r}}-{{\bf s}}|)$, where *U* is a radially symmetric stationary pulse solution of width *a* such that

38 $$ \begin{aligned} U(|{{\bf r}}-{{\bf s}}|)&=\int\limits_{\Upomega_1} w(|{{\bf r}}-{{\bf r}}^\prime|)H(U(|{{\bf r}}^\prime-{{\bf s}}|)-\kappa){\rm d} {{\bf r}}^\prime\\ &\quad+\int\limits_{\Upomega_2} g(|{{\bf s}}-{{\bf s}}^\prime|) H(U(|{{\bf r}}-{{\bf s}}^\prime|)-\kappa){\rm d} {{\bf s}}^\prime\\ &= \int\limits_0^{2\pi}\int\limits_0^a [w(|{{\bf r}}-{{\bf r}}^\prime|)+g(|{{\bf r}}-{{\bf r}}^\prime|)]r^\prime{\rm d} r^\prime{\rm d} \theta, \end{aligned} $$

and

39 $$ \overline{I}(|{{\bf r}}-{{\bf s}}|)=\int\limits_{\Upomega_2} g(|{{\bf s}}-{{\bf s}}^\prime|) H(U(|{{\bf r}}-{{\bf s}}^\prime|)-\kappa){\rm d} {{\bf s}}^\prime. $$

Such a solution represents a continuous topographic map in which an input from ${{\bf s}} \in \Upomega_2$ is mapped to the centre of cortical output activity at the corresponding point ${{\bf s}} \in \Upomega_1$. Stability of the bump (on the fast time–scale of cortical dynamics) can be analysed along identical lines to section ‘Azimuthal Instabilities’, after setting* β* = 0 and taking *w* → *w* + *g*. The corresponding eigenvalues are $\lambda_n=-1+\gamma^{-1}\nu_n$ with |*U*′(*a*)| = *γ* and

40 $$ \nu_n=a\int\limits_{0}^{2\pi} [w(2a\sin(\theta/2)) + g(2a\sin(\theta/2)) ]\cos(n\theta) {\rm d} \theta. $$

Differentiating Eq. (4) with respect to *r* and setting *r* = *a* shows that* ν*_1_ = *γ* and, hence,* λ*_1_ = 0. The existence of a zero eigenvalue reflects the underlying translation symmetry of the two-layer network, which implies that the bump is marginally stable with respect to uniform shifts in space. Thus, the bump will be stable provided that* ν*_*n*_ < *γ* for all *n* ≠ 1. In the following, we assume that there exists a unique stable bump.

We now investigate the stability of the two–dimensional topographic map (on the slow timescale of the input or weights dynamics) by linearising Eqs. (35) and (36) about the homogeneous radially symmetric solution given by Eqs. (38) and (39). First, introducing the perturbations

41 $$ u({{\bf r}}|{{\bf s}},t)=U(|{{\bf r}}-{{\bf s}}|)+p({{\bf r}}|{{\bf s}},t),\quad I({{\bf r}}|{{\bf s}},t)=\overline{I}(|{{\bf r}}-{{\bf s}}|)+q({{\bf r}}|{{\bf s}},t), $$

and expanding to first order in *p*, *q* leads to the linear equations (on setting* η* = 1)

42 $$ \frac{\partial q}{\partial t}=-q({{\bf r}}|{{\bf s}},t)+ \int\limits_{R^2} g(|{{\bf s}}-{{\bf s}}'|) H'(U(|{{\bf r}}-{{\bf s}}'|)p({{\bf r}}|{{\bf s}}',t){\rm d} {{\bf s}}', $$

and

43 $$ p({{\bf r}}|{{\bf s}},t)=q({{\bf r}}|{{\bf s}},t)+ \int\limits_{R^2} w(|{{\bf r}}-{{\bf r}}^\prime|) H^\prime(U(|{{\bf r}}^\prime-{{\bf s}}|)p({{\bf r}}^\prime|{{\bf s}},t) {\rm d} {{\bf r}}^\prime. $$

Using the identity

44 $$ H^\prime(U(|{{\bf r}}-{{\bf s}}|) =\gamma^{-1} \delta(|{{\bf r}}-{{\bf s}}|-a), $$

we can reduce the above linear equations to the form

45 $$ \frac{\partial q}{\partial t}=-q({{\bf r}}|{{\bf s}},t)+ \frac{a}{\gamma}\int\limits_{0}^{2\pi} g(|{{\bf s}}-{{\bf r}}+a{{\bf e}}_{\phi}|) p({{\bf r}}|{{\bf r}}-a{{\bf e}}_{\phi},t){\rm d} \phi, $$

and

46 $$ p({{\bf r}}|{{\bf s}},t)=q({{\bf r}}|{{\bf s}},t)+ \frac{a}{\gamma}\int\limits_{0}^{2\pi} w(|{{\bf r}}-{{\bf s}} -a{{\bf e}}_{\phi}|) p({{\bf s}}+a{{\bf e}}_{\phi}|{{\bf s}},t){\rm d} \phi, $$

with $${{\bf e}}_{\theta}=(\cos \theta, \sin \theta)$$. Defining

47 $$ p_{\theta}({{\bf s}},t)=p({{\bf s}}+a{{\bf e}}_{\theta}|{{\bf s}},t),\quad q_{\theta}({{\bf s}},t)=q({{\bf s}}+a{{\bf e}}_{\theta}|{{\bf s}},t), $$

and setting **r** = **s** + *a* **e**_θ_ in Eq. (46), we find that

48 $$ \begin{aligned} p_{\theta}({{\bf s}},t)&=q_{\theta}({{\bf s}},t)+ \frac{a}{\gamma}\int\limits_{0}^{2\pi} w(a|{{\bf e}}_{\theta}-{{\bf e}}_{\phi}|) p_{\phi}({{\bf s}},t){\rm d} \phi \\ &= q_{\theta}({{\bf s}},t)+ \frac{a}{\gamma}\int\limits_{0}^{2\pi} w(2a\sin([\theta-\phi]/2) p_{\phi}({{\bf s}},t){\rm d} \phi. \end{aligned} $$

We have used the identity

49 $$ |{{\bf e}}_{\theta}-{{\bf e}}_{\phi}|^2=2[1-\cos(\theta-\phi)]=4\sin^2([\theta-\phi]/2). $$

Similarly, setting **r** = **s** + *a* **e**_θ_ in Eq. (45) gives

50 $$ \frac{\partial q_{\theta}}{\partial t}=-q_{\theta}({{\bf s}},t)+ \frac{a}{\gamma}\int\limits_{0}^{2\pi} g(2a\sin([\theta-\phi]/2) p_{\phi}({{\bf s}}+a({{\bf e}}_{\theta}-{{\bf e}}_{\phi}),t) {\rm d} \phi. $$

The neural field perturbations *p*_θ_(**r**, τ) can be related to perturbations of the circular boundary of the activity bump. Let us write the perturbed threshold condition in the form *u*(**r**|**s**, *t*) = κ at **r** = **s** + (*a* + *ρ*(*θ*, *ξ*, *t*))**e**_*θ*_. This yields

51 $$ \begin{aligned} \kappa&=U(a+\rho(\theta,\xi,t))+p({{\bf s}}+(a+\rho(\theta,{{\bf s}},t)){{\bf e}}_{\theta}|{{\bf s}},t) \\ &=U(a)+U'(a)\rho(\theta,{{\bf s}},t)+p({{\bf s}}+a{{\bf e}}_{\theta}|{{\bf s}},t)+{\mathcal O}(\rho^2), \end{aligned} $$

which implies that

52 $$ \rho(\theta,{{\bf s}},t)=\gamma^{-1}p_{\theta}({{\bf s}},t), $$

since *U*(*a*) = *κ* and *U*′(*a*) =  −*γ*. Here* ρ*(*θ*, **s**) represents the radial shift in the* θ* direction of the circular bump boundary centred at* ξ* and for fixed* ξ* corresponds directly to the boundary perturbation considered in section ‘Azimuthal Instabilities’.

Equations (48) and (50) have solutions of the form

53 $$ p_{\theta}({{\bf s}},t)={\rm e}^{\lambda t}{\rm e}^{i{{\bf k}}\cdot {{\bf s}}}P_{\theta}({{\bf k}}),\quad q_{\theta}({{\bf s}},t)={\rm e}^{\lambda t}{\rm e}^{i{{\bf k}}\cdot {{\bf s}}}Q_{\theta}({{\bf k}}), $$

with

54 $$ P_{\theta}({{\bf k}})=Q_{\theta}({{\bf k}})+ \frac{a}{\gamma}\int\limits_{0}^{2\pi} w(2a\sin([\theta-\phi]/2) P_{\phi}({{\bf k}}) {\rm d} \phi, $$

and

55 $$ \lambda Q_{\theta}({{\bf k}})=-Q_{\theta}({{\bf k}})+ \frac{a}{\gamma}\int\limits_{0}^{2\pi} g(2a\sin([\theta-\phi]/2) {\rm e}^{ia{{\bf k}}\cdot ({{\bf e}}_{\theta}-{{\bf e}}_{\phi})}P_{\phi}({{\bf k}}) {\rm d} \phi. $$

Equations (54) and (55) can be analysed further by introducing the Fourier series

56 $$ P_{\theta}({{\bf k}})=\sum_{n \in {{\bf Z}}} P_n({{\bf k}}){\rm e}^{in\theta},\quad Q_{\theta}({{\bf k}})=\sum_{n \in {{\bf Z}}} Q_n({{\bf k}}){\rm e}^{in\theta}. $$

This leads to the discrete set of equations

57 $$ (\lambda+1)Q_n({{\bf k}})=\gamma^{-1}\sum_{n\in {{\bf Z}}}G_{nn^\prime}({{\bf k}})P_{n^\prime}({{\bf k}}), $$

58 $$ P_n({{\bf k}})=\frac{Q_n({{\bf k}})}{1-\gamma^{-1}\mu_n}, $$

with

59 $$ G_{nn^\prime}({{\bf k}})=a\int\limits_0^{2\pi}{\rm e}^{-in\theta}\int\limits_{0}^{2\pi} {\rm e}^{in^\prime\phi}g(2a\sin([\theta-\phi]/2) {\rm e}^{ia{{\bf k}}\cdot ({{\bf e}}_{\theta}-{{\bf e}}_{\phi})}\frac{{\rm d} \phi {\rm d} \theta}{2\pi}, $$

and

60 $$ \mu_n=a\int\limits_{0}^{2\pi} w(2a\sin(\theta/2)) \cos(n\theta) {\rm d} \theta. $$

### Calculation of Eigenmodes

Determining the stability of the two-dimensional topographic map has reduced to the problem of finding the eigenvalues of the infinite-dimensional matrix *G*_*nn*′_(**k**) for $n,n^\prime\in {{\bf Z}}$. It is not possible to do this analytically for general input kernel *g*. However, an explicit solution can be obtained in the limiting case of wide Gaussian inputs such that* σ* ≫ *a* in Eq. (37). We can then carry out a perturbation expansion in *a* ^2^/σ^2^ by writing

61 $$ \begin{aligned} g(2a\sin(\theta/2))&= c{\rm e}^{-a^2\sin^2(\theta/2)/\sigma^2} -\hat{c} \\ &\approx c-\hat{c}-\frac{ca^2}{2\sigma^2}(1-\cos(\theta))+{\mathcal O}(a^4/\sigma^4). \end{aligned} $$

Keeping only lowest order terms, we find that

62 $$ G_{nn^\prime}({{\bf k}})\approx a(c-\hat{c})\int\limits_0^{2\pi}{\rm e}^{-in\theta}\int\limits_{0}^{2\pi} {\rm e}^{in^\prime\phi} {\rm e}^{iak (\cos(\theta-\varphi)-\cos(\phi-\varphi))}\frac{{\rm d}\phi {\rm d} \theta}{2\pi},$$

where ${{\bf k}} = (k,\varphi)$ in polar coordinates. The integrals over* ϕ* and* θ* may now be evaluated using the following Bessel function expansion:

63 $$ {\rm e}^{ika\cos (\theta-\varphi)}=\sum_{m\in {{\bf Z}}}(-i)^mJ_m(ka){\rm e}^{im(\theta-\varphi)}, $$

with *J*_−*m*_ = *J*_*m*_. This gives

64 $$ \begin{aligned} G_{nn^\prime}({{\bf k}})&\approx a(c-\hat{c})\int\limits_0^{2\pi}{\rm e}^{-in\theta}\int\limits_{0}^{2\pi} {\rm e}^{in^\prime\phi} \sum_{m\in {{\bf Z}}}(-i)^mJ_m(ka){\rm e}^{im(\theta-\varphi)}\\ &\quad\times \sum_{m^\prime\in {{\bf Z}}}(i)^{m^\prime}J_{m^\prime}(ka){\rm e}^{-im^\prime(\phi-\varphi)}\frac{{\rm d} \phi {\rm d} \theta}{2\pi}\\ &=2\pi a(c-\hat{c})(-i)^n(i)^{n^\prime}J_n(ka)J_{n^\prime}(ka){\rm e}^{i(n^\prime-n)\varphi}. \end{aligned} $$

Similarly, substituting Eq. (61) into (40) and gives to lowest order

65 $$ \mu_0 \approx \nu_0 -2\pi a (c-\hat{c}), \quad \mu_1 \approx \nu_1 -\pi a c\frac{a^2}{2\sigma^2}, \quad \mu_n \approx \nu_n,\quad n >1. $$

Combining Eqs. (57), (58), and (64) yields a vector equation of the form

66 $$ {{\bf b}}^*({{\bf k}})({{\bf b}}({{\bf k}})\cdot \widehat{{\bf P}}({{\bf k}}))=(1+\lambda)\widehat{{\bf P}}({{\bf k}}), $$

where ^*^ denotes complex conjugate, and

67 $$ \widehat{P}_n({{\bf k}}) =\sqrt{\gamma -\mu_n}P_n({{\bf k}}),\quad b_n({{\bf k}}) =\sqrt{\frac{2\pi a(c-\hat{c})}{\gamma-\mu_n}}(i)^n J_n(ka){\rm e}^{in\varphi}. $$

There are two classes of solution to Eq. (66). If ${{\bf b}}\cdot \widehat{{\bf P}}=0$ then* λ* = −1 and the topographic map is stable with respect to excitation of the corresponding eigenmodes. On the other hand, if ${{\bf b}}\cdot \widehat{{\bf P}}\neq 0$ then $\widehat{{\bf P}}={{\bf b}}^*$ (up to a constant multiplicative factor). Substituting into the Fourier series (56), the resulting eigenmode is of the form $P_{\theta}({{\bf k}}) = P(k,\theta-\varphi)$ with ${{\bf k}}=(k,\varphi)$,

68 $$ \begin{aligned} P(k,\theta)&=\Upgamma \left [\frac{J_{0}(ka)}{\gamma-\mu_{0}}+2\sum_{n\geq 1}(-1)^n\frac{J_{2n}(ka)}{\gamma-\mu_{2n}}\cos(2n\theta)\right.\\ &\quad-\left .2\sum_{n\geq 1}(-1)^n\frac{J_{2n-1}(ka)}{\gamma-\mu_{2n-1}}\sin((2n-1)\theta)\right], \end{aligned} $$

where $\Upgamma$ is an arbitrary amplitude. The corresponding eigenvalue is* λ* = *λ*(*k*) with

69 $$ \lambda(k)=-1+|{{\bf b}}|^2 =-1+\sum_{n\in {{\bf Z}}}{\frac{2\pi a(c-\hat{c})}{\Upgamma-\mu_n}}J_n(ka)^2. $$

For the sake of illustration, suppose that *μ*_*n*_ < *γ* for all $n\in {{\bf Z}}$. This is plausible given Eq. (65) and the conditions on μ_*n*_. Equation (69) implies that if $c<\hat{c}$ then the topographic map is stable since λ(*k*) < 0 for all *k*. On the other hand, if $c>\hat{c}$ such that* λ*(*k*_*c*_) = max_*λk*_(*k*) > 0 then the topographic map is unstable and the fastest growing eigenmodes have the critical wavenumber *k*_*c*_. Recall that* γ* = *ν*_1_. It then follows from Eq. (65) that μ_1_ ≈* γ* and the dominant contribution to the sum in Eq. (69) will arise from the *n* = 1 term, at least away from the zeros of *J*_1_(*ka*). Hence, *k*_*c*_ is approximately given by the point at which the first-order Bessel function attains its global maximum, that is, |*J*_1_(*ak*_*c*_)| = max_*k*_ |*J*_1_(*ak*)|. Numerically one finds that *k*_*c*_ ≈ 3/*a*. Note that the eigenvalues* λ*(*k*), *k* ≠ 0, have an infinite degeneracy that reflects the additional rotation symmetry of the system. That is, all eigenmodes *P*_*θ*_(**k**) with |**k**| = *k* have the same eigenvalue. It follows that the pattern-forming instability will be dominated by some linear combination of eigenmodes lying on the critical circle |**k**| = *k*_*c*_:

70 $$ p_{\theta}({{\bf r}})=\sum_{i=1}^N \left (z_i {\rm e}^{i{{\bf k}}_i\cdot {{\bf r}}}+z_i^*{\rm e}^{i{{\bf k}}_i\cdot {{\bf r}}}\right ) P(k_c,\theta-\varphi_i), $$

where ${{\bf k}}_i=(k_c,\varphi_i)$ and *z*_*i*_ is a complex amplitude. Suppose that each eigenmode can be approximated by the first three terms of Eq. (68) so that

71 $$ P(k_c,\theta)\approx \Upgamma \left[\frac{J_{0}(k_ca)}{\gamma-\mu_{0}}+ \frac{2J_{1}(k_ca)}{\gamma-\mu_{1}}\sin(\theta)- \frac{2J_{2}(k_ca)}{\gamma-\mu_{2}}\cos(2\theta)\right ]. $$

The first term generates an expansion of the bump, the second term generates a uniform shift of the bump, and the third term generates an elongation of the bump (see Fig. 2). In general, we expect the eigenmode (71) to be dominated by the first harmonic term $\sin(\theta)$, since *μ*_1_ ≈* γ*. However, if *μ*_2_ ≈* γ* as well, then there could also be a significant contribution from the term $\cos(2\theta)$. Thus, the spontaneous symmetry breaking mechanism has the potential for generating elongated or oriented bumps. Moreover, since each eigenmode in the sum (70) then represents an elongation in the direction $\varphi_i$ or $\pi/2+\varphi_i$ (depending on the sign of its associated coefficient $z({{\bf r}})=z_i {\rm e}^{i{{\bf k}}_i\cdot {{\bf r}}}+z_i^*{\rm e}^{i{{\bf k}}_i\cdot {{\bf r}}}$), it follows that there is some complicated variation in the preferred bump orientation as **r** varies across the cortex.

### Euclidean Shift-Twist Symmetry and Orientation Preference Maps

The two-dimensional isotropic and homogeneous neural field Eqs. (35) and (36) are equivariant with respect to the product Euclidean group **E**(2) × **E**(2) acting on the space **R** ^2^ × **R** ^2^ according to

72 $$ \begin{aligned} T_{{{\bf d}},{{\bf d}}^\prime}\cdot({{\bf r}},{{\bf s}}) &= ({{\bf r}}+{{\bf d}},{{\bf s}}+{{\bf d}}^\prime), \\ R_{\xi,\xi^\prime}\cdot({{\bf r}},{{\bf s}}) &= (R_{\xi}{{\bf r}},R_{\xi^\prime}{{\bf s}}), \\ R_{\kappa,\kappa^\prime}\cdot({{\bf r}},{{\bf s}}) &= (R_{\kappa} {{\bf r}},R_{\kappa^\prime} {{\bf s}}), \end{aligned} $$

where *R*_*ξ*_ **r** denotes the planar rotation of **r** through an angle* ξ*, and *R*_κ_ = *R*_ ± _ with *R*_ ± _(*x*, *y*) = (*x*,  ± *y*). The corresponding group action on the neural fields *u*, *I* is

73 $$ \begin{aligned} T_{{{\bf d}},{{\bf d}}^\prime}(u({{\bf r}}|{{\bf s}}),I({{\bf r}}|{{\bf s}})) &= (u({{\bf r}}-{{\bf d}}|{{\bf s}}-{{\bf d}}^\prime),I({{\bf r}}-{{\bf d}}|{{\bf s}}-{{\bf d}}^\prime)),\\ R_{\xi,\xi^\prime}(u({{\bf r}}|{{\bf s}}),I({{\bf r}}|{{\bf s}})) &= (u(R_{-\xi}{{\bf r}}|R_{-\xi^\prime}{{\bf s}}),I(R_{-\xi}{{\bf r}}|R_{-\xi^\prime}{{\bf s}})),\\ R_{\kappa,\kappa^\prime}(u({{\bf r}}|{{\bf s}}),I({{\bf r}}|{{\bf s}})) &= (u(R_{\kappa}{{\bf r}}|R_{\kappa^\prime}{{\bf s}}),I(R_{\kappa}{{\bf r}}|R_{\kappa^\prime}{{\bf s}})). \end{aligned} $$

Equivariance means that if (*u*, *v*) is a solution of the neural field equations then so is $\eta\cdot (u,v)$ for all $\eta \in {{\bf E}(2)}\times {{\bf E}(2)}$. In other words, the two-dimensional network has both translation and rotation/reflection symmetries. An isotropic and homogeneous equilibrium solution of the form $u({{\bf r}}_2|{{\bf r}})=U(|{{\bf r}}_2-{{\bf r}}|),I({{\bf r}}_2|{{\bf r}})=\overline{I}(|{{\bf r}}_2-{{\bf r}}|)$ then explicitly breaks the symmetry group from ${{\bf E}(2)}\times {{\bf E}(2)}\rightarrow {{\bf E}(2)}$ with **E**(2) having the group elements *T*_**d**_ = *T*_**d**_, **d**,  *R*_ξ_ = *R*_ξ,ξ_ and *R*_κ_ = *R*_κ,κ_. As we have shown above, the homogeneous equilibrium solution can undergo a pattern-forming instability that spontaneously breaks the remaining Euclidean symmetry.

The Euclidean symmetry of the full Eqs. (35) and (36) also manifests itself in a reduced form after linearising about the homogeneous solution. That is, the linear equations (48) and (50) are equivariant with respect to the so-called shift-twist action of the Euclidean group **E**(2) on the space **R** ^2^ × *S* ^1^ [10, 11]:

74 $$ \begin{aligned} T_{{\bf d}}\cdot({{\bf r}},\theta) &= ({{\bf r}}+{{\bf d}},\theta), \\ R_{\xi}\cdot({{\bf r}},\theta) & = (R_{\xi}{{\bf r}},\theta+{{\bf s}}), \\ R_{\kappa}\cdot({{\bf r}},\theta) & = (R_{\kappa} {{\bf r}},- \theta), \end{aligned} $$

where *R*_κ_(*x*, *y*) = (*x*,  −*y*). The corresponding action on the fields *p*_θ_(**r**) and *q*_*θ*_(**r**) is

75 $$ \begin{aligned} T_{{\bf d}}\cdot (p_{\theta}({{\bf r}}),q_{\theta}({{\bf r}})) &= (p_{\theta}({{\bf r}}-{{\bf d}}),q_{\theta}({{\bf r}}-{{\bf d}})),\\ R_{\xi}\cdot (p_{\theta}({{\bf r}}),q_{\theta}({{\bf r}})) &= (p_{\theta-\xi}(R_{-\xi}{{\bf r}}),q_{\theta-\xi}(R_{-\xi}{{\bf r}})), \\ R_{\kappa}\cdot (p_{\theta}({{\bf r}}),q_{\theta}({{\bf r}})) &= (p_{-\theta}(R_{\kappa}{{\bf r}}),q_{-\theta}(R_{\kappa}{{\bf r}})). \end{aligned} $$

**Fig. 6:**
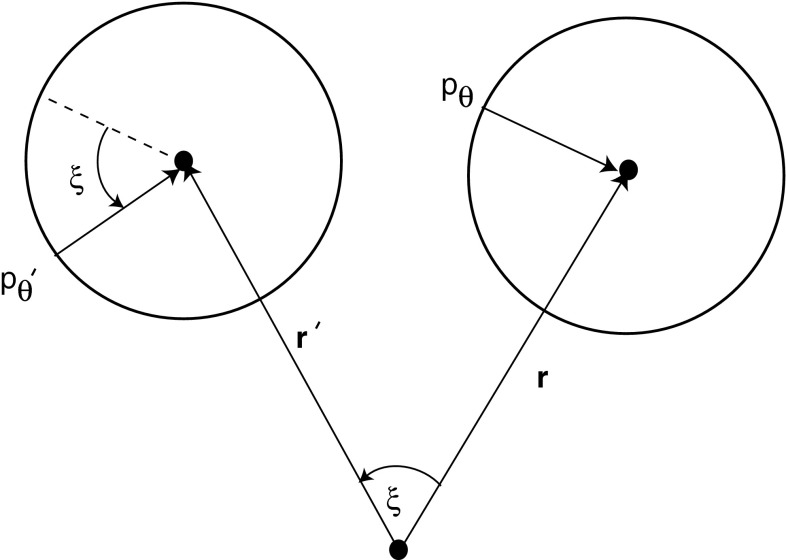
Action of a rotation by $\xi:\,p_{\theta}({{\bf r}}) \rightarrow p_{\theta^\prime}({{\bf r}}^\prime)$ where (**r**′, *θ*′) = (*R*_ξ_ **r**, *θ* + *ξ*). Here **r** represents the position of the centre of a two-dimensional bump and *p*_*θ*_ represents the perturbation of steady-state activity at a point θ on the boundary of the bump

It can be seen that the rotation operation comprises a translation or *shift* of the angle* θ* to* θ* + **s**, together with a rotation or *twist* of the position vector **r** by the angle **s**. This is illustrated in Fig. 6. One of the consequences of the underlying Euclidean symmetry is that the associated eigenfunctions form irreducible representations of the shift-twist group action [10, 11]. This explains why the eigenmodes *P*_θ_(**k**) have the basic structure given by equation (68), with the angular variable* θ* coupled to the direction of the wavevector **k**.

The emergence of elongated cortical activity bumps reflects a corresponding elongation in the receptive field properties of cortical cells. Hence, the spontaneous symmetry breaking of the topographic map between LGN and visual cortex provides a possible mechanism for the development of orientation preference columns in primary visual cortex. The variation in orientation with cortical position then determines the corresponding orientation preference map. Interestingly, a recent statistical analysis of orientation preference maps in primates indicates that there are correlations between the direction of the topographic axis joining pairs of columns with similar orientation preferences and their common orientation [34]. Thus, the orientation preference map exhibits a form of rotational shift-twist symmetry as predicted from our analysis of two-dimensional topographic maps. Numerical simulations of a feature-based dynamical spin model has led to the suggestion that such a symmetry could help to stabilise the emerging orientation preference map with its associated set of pinwheels [34]. As previously shown by Wolf and Geisel [55], in the absence of such a coupling, the pinwheels typically annihilate in pairs. Hence, in order to maintain pinwheels, either development has to be stopped or one has to introduce inhomogeneities that trap the pinwheels.

## Discussion

In this review, we have described John G Taylor’s work on neural bubble dynamics [48], as well as various extensions that have revealed a rich repertoire of 2D network dynamics, including breathers [15, 20, 39], multiple bumps [32], labyrinthine structures [16, 39], rotating waves [21], spiral waves [26, 29, 31, 43], and travelling spots [15, 16]. Traditionally, in vitro methods for studying the generation and propagation of electrical activity in networks of neurons have involved removing a thin vertical slice of brain tissue [13, 23, 40, 42], which makes it difficult to observe the various forms of 2D dynamics predicted by neural field theory. However, recent studies of tangential slices have demonstrated that various phenomena predicted by neural field theory are also observed in living tissue [26, 57]. Interestingly the type of neural field models discussed here (and their localised solutions) has been adopted as a formal framework for thinking about embodied cognitive dynamics by Gregor Schöner and colleagues, see for example [28]. Initially, this work focused on developing a theory of how eye movements are planned, though has since been expanded to a variety of topics including motor planning and visuospatial cognition. Moreover, the same style of conceptual modelling has proven fruitful in developing a theory of cognitive robotics [18]. There is clearly still much to be gained by the mathematical study of neural field models along the lines developed by John G Taylor and others.

## Appendix

The double integral in (3) can be calculated using Fourier transforms and Bessel function identities [20]. First, express *w*(*r*) as a 2D Fourier transform using polar coordinates:

76 $$ w(r) = \frac{1}{2\pi}\int\limits_{{\mathbb{R}}^2} {\rm e}^{i{\bf r}\cdot{\bf k}}\widehat{w}({\bf k}) {\rm d} {\bf k}=\frac{1}{2\pi}\int\limits_0^\infty\left(\int\limits_0^{2\pi}{\rm e}^{ir\rho\cos \theta}\widehat{w}(\rho) {\rm d} \theta\right) \rho {\rm d} \rho, $$

where $\widehat{w}$ denotes the Fourier transform of *w* and **k** = (*ρ*, *θ*). Using the integral representation

77 $$ \frac{1}{2\pi}\int\limits_0^{2\pi}{\rm e}^{ir\rho\cos \theta} {\rm d} \theta=J_0(\rho r), $$

where *J*_0_ is the Bessel function of the first kind, we express *w* in terms of its Hankel transform of order zero,

78 $$ w(r)=\int\limits_0^\infty \widehat{w}(\rho)J_0({\rho r})\rho {\rm d} \rho, $$

which, when substituted into Eq. (4), gives

79 $$ U(r)=\int\limits_0^{2\pi}\int\limits_0^{\Updelta} \left(\int\limits_0^\infty \widehat{w}(\rho)J_0(\rho |{{\bf r}}-{{\bf r}}'|)\rho {\rm d} \rho\right) r^\prime {\rm d} r^\prime {\rm d} \phi^\prime. $$

Reversing the order of integration and using the addition theorem

80 $$ J_{ 0}\left(\rho\sqrt{r^2+{r^\prime}^2-2rr^\prime\cos\phi^\prime}\right) =\sum_{m=0}^{\infty}\epsilon_mJ_m(\rho r)J_m(\rho {r^\prime}) \cos m\phi^\prime, $$

where $\epsilon_0=1$ and $\epsilon_n=2$ for *n* ≥ 1, we thus obtain (10), using the identity $J_1(\rho \Updelta)\Updelta=\rho \int_0^{\Updelta} J_0(\rho r^\prime) r^\prime{\rm d} r^\prime$.
